# Orf (ecthyma contagiosum)

**DOI:** 10.11604/pamj.2021.38.322.29033

**Published:** 2021-04-01

**Authors:** Konstantina Mavridou, Maria Bakola

**Affiliations:** 1 Department of Skin and Venereal Diseases, University of Ioannina, University Hospital of Ioannina, Ioannina, 45110, Greece,; 2 Derviziana Primary Health Center, Ioannina, Greece

**Keywords:** Orf, ecthyma contagiosum, parapox virus

## Image in medicine

Orf, also known as ecthyma contagiosum, is a parapox virus infection endemic among sheep and goats that can be transmitted to humans, particularly to farmers, sheepherders, butchers and veterinarians. Although it’s a self-limiting infection, complications may be seen such as ocular involvement, erythema multiforme, extensive lesions in patients with atopic dermatitis and secondary bacterial infection. A 29-year-old man with no past medical history, presented to his primary care physician with a well-circumscribed, non-pulsating growth at the proximal phalanx of his right thumb. A carefully taken history revealed that this lesion had appeared three days ago as a red nonhealing papule. The patient reported no history of fever, pain or itching at the site. He mentioned exposure to sheep and goats since he was a shepherd. On examination, the lesion size was approximately 1.5cm in diameter and appeared as a nodule with a red centre surrounded successively with a white ring and then a red halo resembling as a target lesion. No other lesions and no lymphadenopathy were noted. Blood samples were normal and showed no signs of infection. The clinical observations and medical history were compatible with the virus infection Orf. The patient was seen at weekly intervals. The lesion was managed conservatively and no specific therapy was undertaken, other than topical disinfectants. After four weeks the lesion had completely healed without a scar.

**Figure 1:**
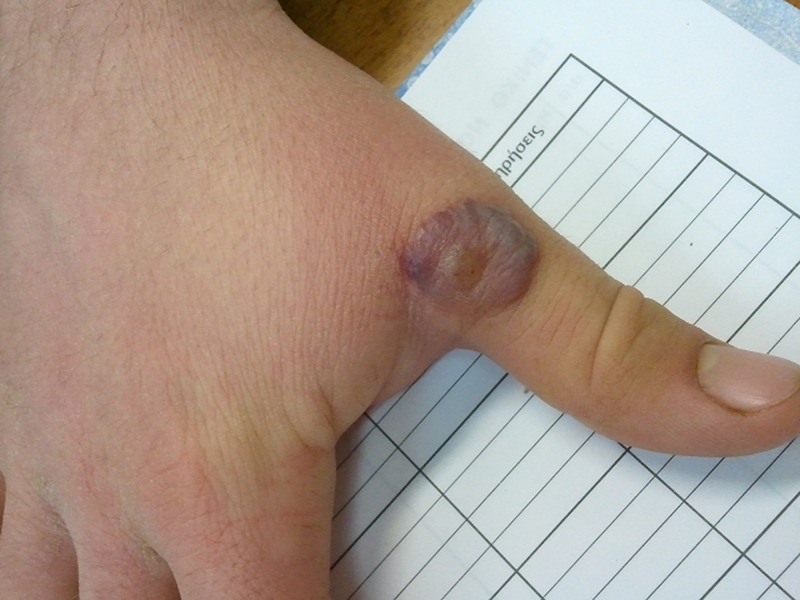
orf (ecthyma contagiosum)

